# An integrative approach to develop computational pipeline for drug-target interaction network analysis

**DOI:** 10.1038/s41598-018-28577-6

**Published:** 2018-07-06

**Authors:** Ankush Bansal, Pulkit Anupam Srivastava, Tiratha Raj Singh

**Affiliations:** Department of Biotechnology and Bioinformatics, Jaypee University of Information Technology, Waknaghat, 173234 Solan, HP India

## Abstract

Understanding the general principles governing the functioning of biological networks is a major challenge of the current era. Functionality of biological networks can be observed from drug and target interaction perspective. All possible modes of operations of biological networks are confined by the interaction analysis. Several of the existing approaches in this direction, however, are data-driven and thus lack potential to be generalized and extrapolated to different species. In this paper, we demonstrate a systems pharmacology pipeline and discuss how the network theory, along with gene ontology (GO) analysis, co-expression analysis, module re-construction, pathway mapping and structure level analysis can be used to decipher important properties of biological networks with the aim to propose lead molecule for the therapeutic interventions of various diseases.

## Introduction

Analysis of protein interaction network for targets of FDA approved drugs and genes related to disease in OMIM revealed that most drug targets are not even closer to the genes specifically involved in the disease and hence reflects the lack of selectivity in traditional drugs towards the genetic cause^1^. Besides, biasness of literature-mined interaction sets towards well-known proteins, dependence of current approach on target profile similarity or identification of shortest path between drug targets in the interactome has proved to be less efficient in the analysis of relationship between drugs and disease^2,3^.

However, an interdisciplinary approach like the ones used by *Albert-László Barabási Group* has reflected its efficiency to predict novel targets and other uses of the existing drugs through network-driven knowledge^4–8^. In addition, recent findings have demonstrated that genes associated with a disease, tend to cluster in a disease oriented module and represent a connected sub-network within the interactome^9–11^. Many online databases and network approaches have been developed to handle drug-target interaction such as prediction of drug target interaction by integrating protein sequences and drug chemical strcutures^12^, network construction on the basis of heterogeneous biological data^13^, non-coding RNAs and drug targets based networks^14^, drug target interaction prediction models^15,16^, rotation forest-based drug target prediction^17,18^, and drug target prediction using deep neural networks^19^. This led us to think, that for a drug to be efficient enough to cure a disease, it must target proteins within or in the immediate vicinity of the disease module formed by the well-associated genes^20,21^. Hence, to understand therapeutic action of drugs at different levels of biological organization, we developed an unsupervised and unbiased network-driven framework to come-up with a drug-disease proximity measure that would help us to quantify the therapeutic effect of drugs.

In this study, we selected Picroliv to understand of the context within which drug-target interactions at molecular level can lead to distal effectors in a process that result in adverse phenotypes at the organ and organismal levels. Picroliv, is one of the active compounds yielded by underground parts of *Picrorhiza kurroa*, growing at elevations of 3,000–5,000 meters. It is usually a mixture of kutkoside and picroside-I in 1:1.5 ratio^22^. While the other major product synthesized by the underground part is kutkin composed of picroside-I and picroside-II^23^.

The active principal component of *Picrorhiza kurroa* is kutkin comprehend kutkoside and the iridoid glycoside picrosides I, II. Picroside I, also known as 6′*-O-cinnamoylcatalpol*, forms a stable mixture with kutkoside to form kutkin^24^. Another isolated catalpol derivative, identified as *6-O-vanilloylcatalpol*, was named Picroside II^23^. Traditionally picrorhiza has been used to treat disorders of the liver and upper respiratory tract, dyspepsia, chronic diarrhea, and scorpion sting. Studies on Picrorhiza show its crucial role in restoring the depleted glutathione levels in rats infected with malaria^22,24^. Further studies on picrorhiza reveal its anti-lipid peroxidative effect^25^. Recent studies show that Picroside II plays a critical role in preventing the alterations that take place in I/R injury^26,27^. Although the anticancerous activity of picrorhiza has been exploited, its exact molecular mechanisms of actions and related pathways and targets remains poorly understood^28^.

To achieve the desired therapeutic effect while reducing the risk of unpropitious conditions, with a known drug, it is imperative to identify the neighborhood of these targets within which they have their action^29^. Consequently, using information from known drug target and creating networks of associated target proteins; we can understand how drugs can have beneficial as well as pernicious consequences^10^. Based upon these observations, relevant drugs for specific disease could be filtered out to provide only the beneficial population of drugs to the patients.

To decipher the regulatory interactions and underlying mechanistic behavior of picrorhiza, a target-pathway network re-construction was performed to discover the relationship between the drug and its relevant targets and pathways. Construction and analysis of such intricate network not only requires the basic concepts of network biology but also an understanding of how the interaction between drug and its relevant target determines regulation of various phenotypic characters in a diseased state. Besides the direct consequences of the interaction between drug and its target, drug action also depends on the consequences within the physiological system. Therefore a holistic approach is required to deal with drug-target interaction network for the selection of putative drug candidates.

As stated earlier, integration of concepts from various fields can help to reach the best solution for a given problem. Hence, we integrated advanced application of computational and experimental information through literature based support in our work to build networks for analyzing drug action and to develop poly-pharmacology for complex diseases and predict therapeutic efficacy and adverse event risk for individuals prior to commencement of therapy. In this study, we demonstrate a systems pharmacology pipeline and discuss how the network theory in combination with gene ontology (GO) analysis, co-expression analysis, module re-construction, pathway mapping and structural analysis can be used to decipher important properties of biological networks.

## Results and Discussion

To acquire holistic view through empirical data, literature mining was performed to identify known targets for Picroside I, II, III, and IV. Unlike P-I and P-II, no target was identified for P-III and P-IV which led us to drop them for further analysis. The reason for such outcome can be imparted to its inability to cross the blood-brain-barrier^30^. Further, to uncover unknown drug targets those are yet to be verified experimentally, mapping of P-I and P-II structures was done against protein/receptor library through PharmMapper with threshold limited to 30^31^. Combined results from literature mining and PharmMapper, showed the presence of targets common to both and hence were categorized as primary targets while the ones found only in PharmMapper were categorized as secondary targets for downstream analysis.

To further address the question that whether targets taken as secondary are appropriate or not, we retrieved top ten co-expressed genes by considering primary targets as query dataset based on confidence score. Selected nodes were then considered for degree distribution with betweenness centrality, which would state the importance of genes with respect to their association with involved pathways. In this analysis, prioritization of node was done based on *k* and *B*_*c*_ correlation. The node size reflects the association score, i.e., more the association score bigger will be the node and vice-versa.

Genes selected through co-expression analysis were combined together for P-I and II separately and gene ontology analysis was performed using GORILLA to identify the role of target association genes in Biological processes, Molecular functions and Cellular components^32^. Based on *p*-value, association type weak or strong was indicated, which further referred based on well-compiled GO databases. To further understand the role of primary targets we performed the docking analysis using PatchDock server, which checks various conformations and suggests the best one (Table 1). Additionally, we performed the PatchDock analysis with other FDA-approved drugs available in the market to find out the best drug based on molecular interactions in selected targets against P-I, P-II and other drugs^33^. Prioritized targets were cross-checked with literature and found to be key player in carcinogenesis and therefore their role in various malignancies was found to be crucial.

**Table 1:** Low atomic contact energy (ACE) and high geometric shape complementarity score can give an idea of the best target for given ligand and hence can be used for screening targets of ligand.

PDB ID	Uniplot	Score	Area	ACE	Ligand Transformation
**(A)**					
1BL4	FKB1A	1328	451.7	−346.89	0.29948 −0.52512 −2.66923 20.02024 12.12580 7.65261
1DB1	VDR	2190	396.9	−402.49	2.24124 −1.12599 2.08615 −5.02815 24.92850 41.97212
1ICE	CASP1	1290	490.5	−456.81	3.10960 −0.49380 −0.04148 32.33943 52.00560 4.29316
1NMS	CASP3	1624	387.5	−378.14	−0.03535 0.04862 −2.03367 25.21819 13.04502 −9.84853
1PW6	IL2	1720	416.1	−439.09	2.19306 −0.53308 −2.49690 88.10656 30.51603 35.60994
1NXK	P49137	1486	386.8	−484.19	0.26065 1.01573 0.40363 90.77334 9.18137 30.57505
1TFG	TGFB2	3130	346.8	−418.27	0.08687 −0.05031 1.92449 4.06230 37.50500 15.50360
1MQ6	FA10	1300	375.8	−459.61	2.51453 −0.37431 −2.92742 48.50947 3.03372 38.24805
2PE1	PDPK1	1896	461.9	−562.56	−1.77023 0.24687 −2.06069 6.90130 65.74122 28.98557
2RGS	Q16539	1568	455.9	−614.34	2.30613 −1.44196 1.32929 37.41191 −15.10245 31.61810
3C4C	BRAF1	1472	427.3	−488.98	−2.95557 0.19922 −0.83340 −13.00394 17.30278 −0.55134
3FV8	P53779	1366	450.6	−429.62	−1.18766 0.46462 2.66234 −24.40976 −7.18411 1.10192
**(B)**					
1BL4	FKB1A	1950	319.5	−341.15	−1.80502 −1.44915 −1.71611 6.51155 15.99134 27.83651
1BMQ	CASP1	2334	468.1	−494.43	0.86276 0.14930 2.82708 46.73998 50.95963 1.73014
1DB1	VDR	3352	454.5	−392.19	2.55973 −1.10036 0.31187 30.63651 19.85716 62.65453
1GS4	ANDR	1850	363.7	−386.21	−1.81837 0.56780 2.82529 9.23109 9.66646 8.71106
1IG1	DAPK1	1226	427.5	−459.77	−0.88438 0.47386 −0.32604 20.06074 25.86862 24.02309
1KV2	Q16539	1502	451.9	−526.44	−2.86728 −0.26009 0.78648 −2.20249 17.37292 15.16249
1PMN	MK10	2156	474.4	−480.14	0.60624 −0.97594 3.08589 30.23789 2.45222 17.30323
1PY2	IL2	1478	386.5	−431.36	3.13434 0.15769 −1.67019 16.50101 11.89488 74.45355
1RHJ	CASP3	2770	391.2	−326.53	−2.38764 0.21897 −0.92565 −116.92443 16.34730 80.13059
1S9J	MP2K1	1272	378.3	−462.76	−0.07504 0.18569 −2.51140 45.68630 56.41451 15.15250
1NXK	P49137	1958	476.7	−456.24	−2.84526 −0.79813 1.53759 129.57735 31.92929 56.63747
2JRI	FA10	2920	448.1	−368.14	−0.87237 −1.09567 1.46741 −11.67501 −17.26502 6.80250
2PEI	PDPK1	1660	445.9	−547	−0.59030 0.87216 0.49408 −2.14150 55.95997 24.91314
2YXJ	Q07817	1612	421.3	−462.2	0.41523 −0.28987 −2.46397 9.62220 −25.11701 −2.64235

(A) Docking results of various targets considered for Picroside-I from PatchDock v1.3-beta. (B) Docking results of various targets considered for Picroside-II from PatchDock v1.3-beta.

**Figure 1:**
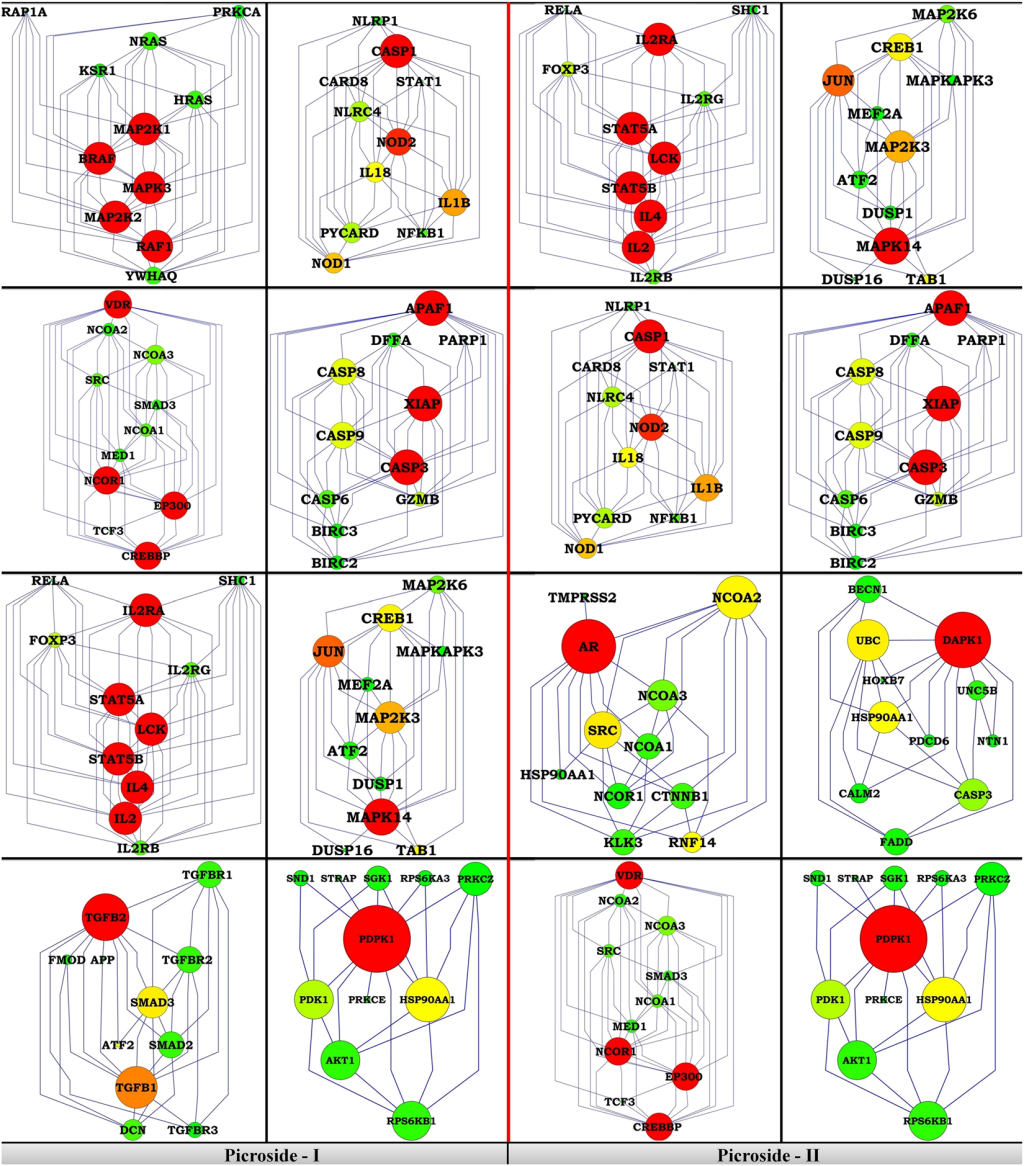
Module wise classification of reconstructed sub-networks (**A**) Picroside – I targets (**B**) Picroside –II targets.

**Figure 2:**
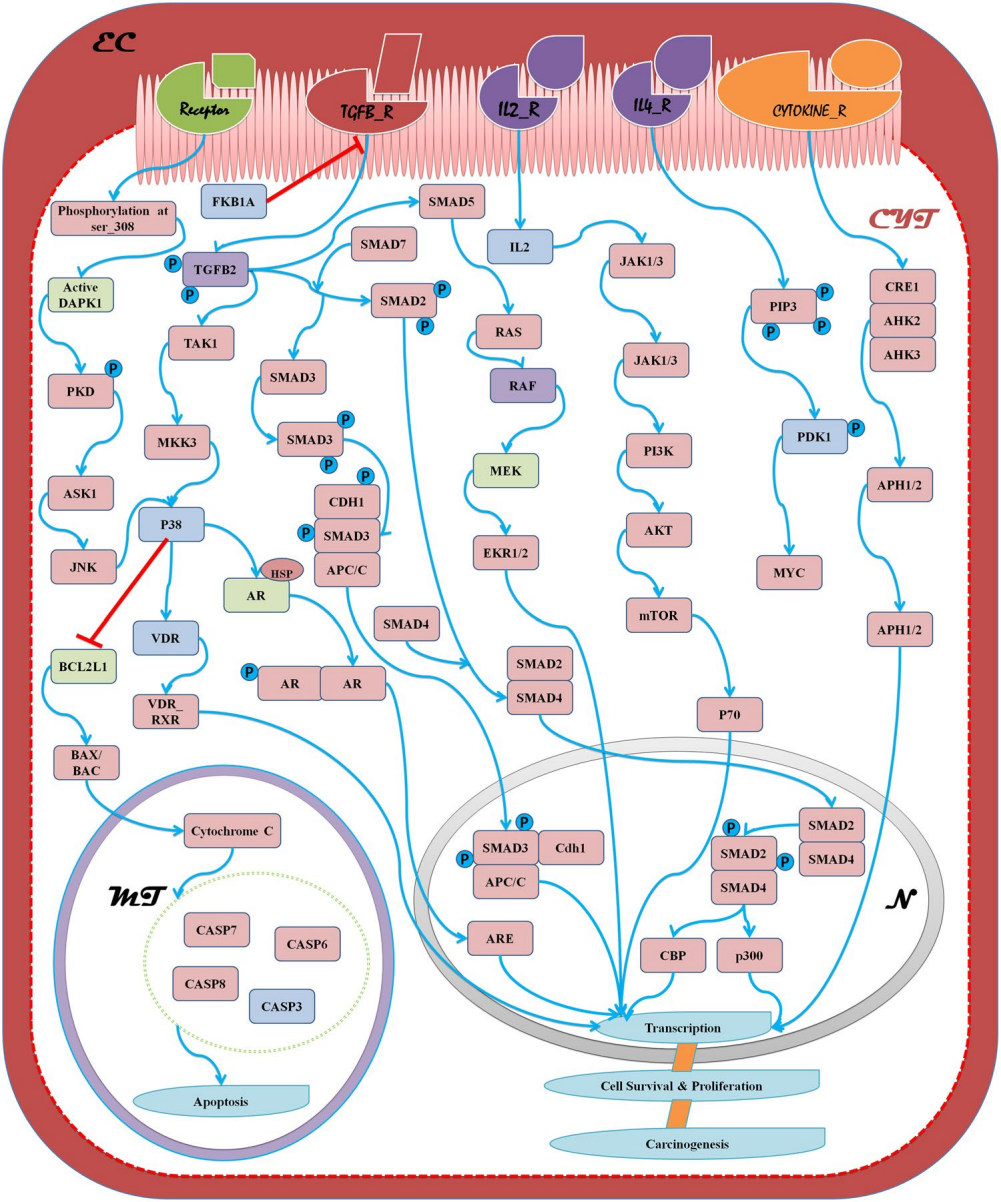
Holistic pathway using network reconstruction approach to represent Death-associated protein kinase 1 (DAPK1), Transforming Growth Factor Beta (TGFβ) signaling, Interleukin (IL) 2, 4 signaling and cytokine signaling for apoptosis and carcinogenesis pathway differentiation.

Primary targets were considered for module definition and tried to converge on pathways on the basis of co-expression based association score. Genes were highlighted using different colors and size, where red color represents the association between degree and betweenness centrality of nodes and node size shows the co-expression association between the interacting nodes (Fig. 1). Mapping of these modules on pathways was performed through pathway reconstruction (Fig. 2).

### Pathway Analysis

For drug-target interaction network we have considered literature mining techniques, scoring functions on the basis of co-expression modules derived from cancer and KEGG database as reference for giving a support factor for holistic network visualization by using Reactome Pathway Database and Pathway Interaction Database. The pipeline presented is based upon working modules where we have compiled the information through the stepwise procedures and outcome of one step can be used as an input for the next step. At few points cross validation is also being applied to present the refined information to the further steps. Pipeline is being verified through all the available data sets for the analysis and finally the robust one is being proposed. We have broadly explored the possible routes and diversion points on the basis of node involvement in networks and data is being generated from standard pathways available. Pathway analysis revealed that genes are distributed in pathways associated with various diseases such as, Cancer associated signaling, Hepatitis–B, Human T-cell leukemia virus type 1 (HTLV-I) Infection, Tuberculosis, Influenza A, Thyroid Hormonal Signaling Pathway and many more. But careful evaluation and mapping on the Kyoto Encyclopedia of Genes and Genomes (KEGG) pathways using combined score resulted association of maximum number of genes with cancer associated signaling, viz. receptor based Death-Associated Protein Kinase 1 (DAPK1) activation, Transforming Growth Factor Beta (TGFβ) signaling, Interleukin (IL) 2, 4 signaling and cytokine signaling. All these signaling cascade results in transcriptional activation and leads to carcinogenesis (Fig. 2).

### Death-Associated Protein Kinase mediated signaling

Calcium/calmodulin-dependent serine/threonine kinases (CDK) play a major role in activation of various signaling via regulating apoptotic pathways^34^. Such kind of activation helps in stimulation of induction of autophagy through JNK regulation as shown in Fig. 2. BRAF1 targeted by both picroside derivatives for inhibiting pathway module as shown in Table 1.

### Transforming growth factor beta mediated signaling

TGFβ signaling acts as crucial regulator in various apoptotic and proliferative pathways. The signaling includes binding to Transforming Growth Factor Beta Receptor TGFBβ-II which further initiates formation of SMAD complex and its phosphorylation ultimately resulting in transcriptional activation of various genes in nucleus^35^. FKB1A, CASP1, CASP3 and TGFB2 targeted by P-I and P-II for blocking TGFβ mediated signaling (Table 1).

### Interleukin Mediated Signaling

IL-2 and IL-4 one of the types of promotes differentiation and proliferation of T helper 2 (TH2) cells and the synthesis of immunoglobulin E (IgE)^36^. Generally, it activates mitogen-activated protein kinase (MAPK), phosphoinositide 3-kinase (PI3K), signal transducers and activators of transcription (STAT), and mammalian target of rapamycin (mTOR) signaling modules, leading to both mitogenic and anti-apoptotic signals^37^. IL2 considered as target for inhibition for inhibiting interleukin mediated singaling (Table 1).

### Cytokine Mediated Signaling

Cytokines plays critical role in the regulation of a various normal functions ranging from cellular proliferation, differentiation and survival to specialized cellular functions enabling host resistance against pathogens. Also, release of cytokines in response to inflammation, immunity or infection can supress cancer development and progression^38^. The JAK-STAT pathway trigerred by cytokines to achieve their ultimate goal can be thought of promising way for cancer therapy in humans^38^. MAPKs acts as central points for target inhibition due to hyperphosphorylation events. Hence, considered as inhibition of proliferative pathways.

**Figure 3:**
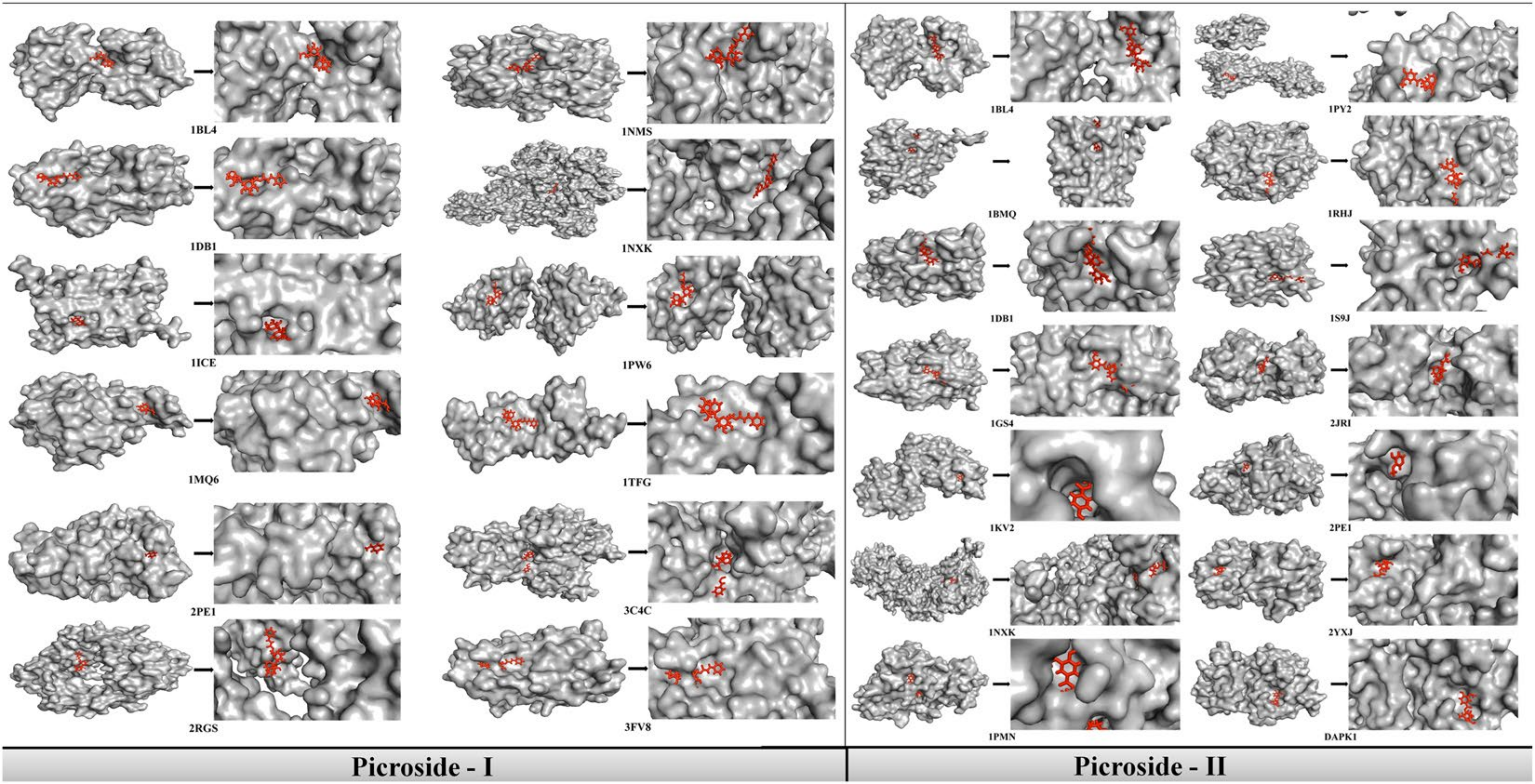
Structural representation of docking result for (A) Picroside-I and targets listed in Table 1A and B) Picroside-II and targets listed in Table 1B. The structural information given as output from PatchDock helps in deciphering the binding site of our ligand with the targets. With the help of parameters listed out in Table 1 we can filter out the best targets and infer their structural interactions.

On the basis of reconstructed pathway, key nodes were selected to perform structural study using PatchDock server. Docking of P-I and P-II was performed and found that picrosides can be used as active inhibitory molecule for cancer treatment as it targets at multiple level which is evident from Fig. 3 and data presented in Table 1. But, there is need to prioritize contender on the basis of personalized gene expression of candidate targets in patients. Picroside derivatives combinely plays a crucial role in inhibition of BRAF, FKB1A, CASP1, CASP3, TGFB2, IL2 and MAPKs through various signaling routes and therefore, can be considered as potent inhibitory molecule for further experimental analysis.

Conclusively, our study presents a novel path to trace down the potential targets and propose them either for treating multiple diseases or for combinatorial therapy by identifying the exact course of disease transmission. It is anticipated that our network based drug-target interaction analysis protocol will assist computational biologists to look for similar patterns in other disease targets and biomedical scientists to design new therapeutic interventions based upon these findings.

## Conclusions

Understanding of regulatory mechanism and subsequent effect on phenotypic level can not be dechiphered through individual genes only, but needs to include coordination of set of genes or gene groups. Hence, there is a need to study combinatorial effects of drugs by targeting multiple triggring points at same instance. With the aid of presented pipeline, biologists can infer key points involved in dysregulation of a particular mechanism given a medicinally important molecule using network-based perspective. For instance Picroside derivatives thought as medicinally important yet have not been broadly investigated for cancer treatment. Our study reveals key markers targeted by picroside derivatives through integration of data mining and network based approaches. The same revealation was found through computational molecular interactions and selected targets can act as potential markers for experimental validation.

## Methods

**Figure 4:**
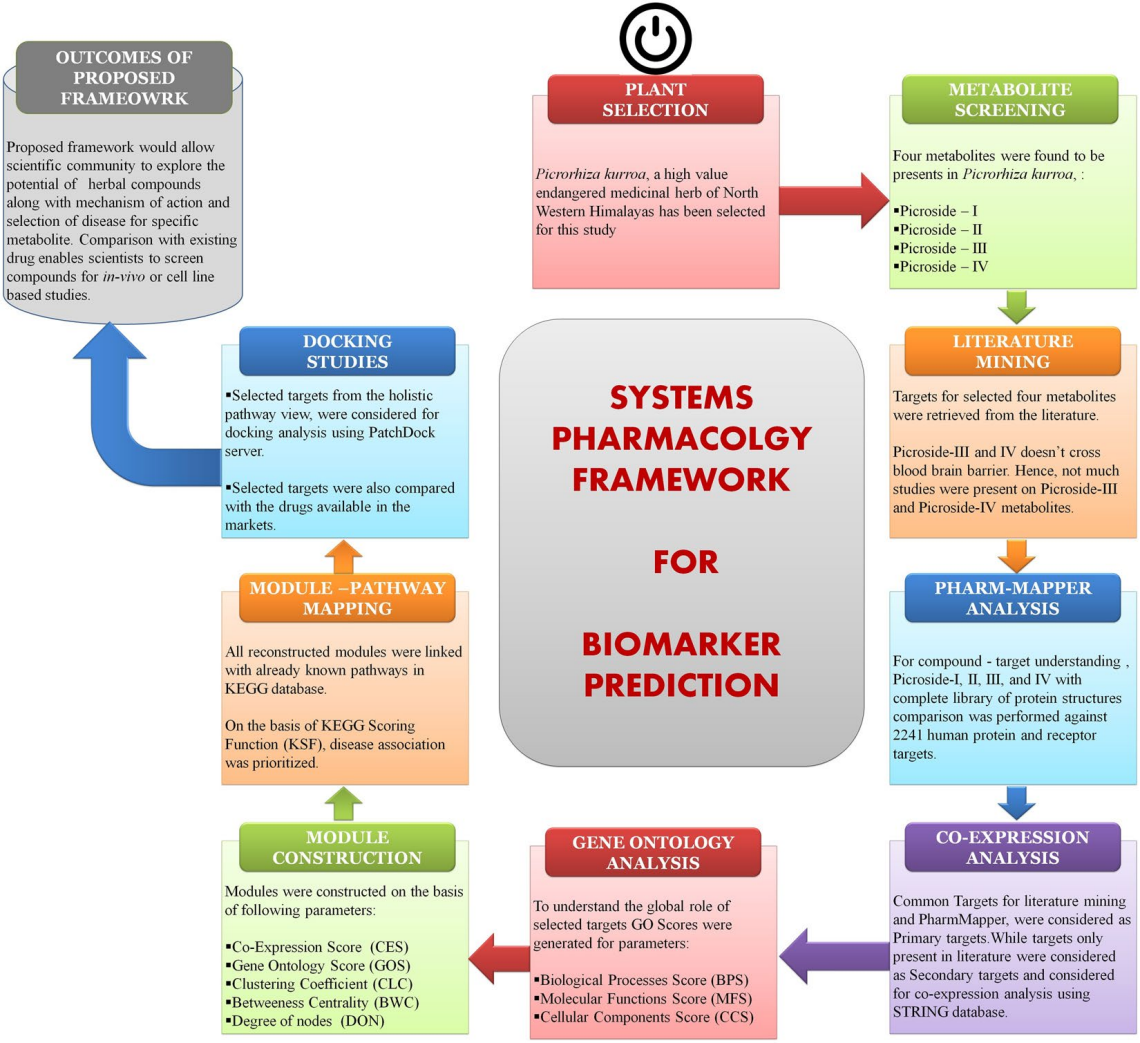
Systems Pharmacology framework for the identification and analysis of biomarkers through various modules viz. medicinal plant selection, metabolite screening, literature mining, pharmMapper analysis, coexpression analysis, gene ontology analysis, module construction, module-pathway mapping and docking study to screen out potential drug targets.

Complex chemical composition of the metabolic compounds found in medicinal herbs makes the understanding of therapeutic mechanism of action arduous. However, to clarify its mechanism of action at molecular level with an aim to know its usefulness in treating disorders, one has to have not only a deep insight into the molecular mechanism but also should opt a systematic approach to aid precise identification of therapeutic target. To achieve the same in this pipeline, literature mining of metabolites along with target network analysis was performed under systems pharmacology framework. Schematic workflow is shown in Fig. 4.

### Literature Mining

With the advancement in scientific era, the information generated in the form of research articles being published in number of journals, is increasing at rapid rate and hence becomes a cumbersome task for a researcher to keep track of relevant literatures from MEDLINE manually. To make the task of information retrieval (IR) much easier, PubMed search engine was used to find all the hits with the query keywords like Picrorhiza (224 hits), Picroside (103 hits) Picroside-I (45 hits) and Picroside-II (78 hits). Screening for Picroside-III and Picroside-IV was also performed in similar fashion. Compiled scoping document of literature mining is available in Supplementary File 1.

### Target Prediction

With the purpose of cross checking the P-I, P-II, P-III, and P-IV interaction with *Homo sapiens* known targets, we downloaded the 2D structure of picroside derivatives from the PubChem library. Further, the downloaded structures were given as an input for PharmMapper^31^. PharmMapper is a web server to predict therapeutic candidate drug targets for small molecule provided as query. To dug out the possible picroside interaction, score for candidate targets was performed by setting the parametric values of 2241 for human targets and a maximum number of 300 reserved matched targets were considered and all other parameters with default values.

### Common Target Identification

A comparative analysis was performed between the targets retrieved from literature and PharmMapper in order to predict the verified target for further consideration of the same as a potential biomarker for various diseases. Targets common to both analysis were considered to have direct interaction for inhibition and therefore are called Primary Targets (PT) in our study. However, the targets that were present in literature and found to be affected but not present in the PharmMapper analysis were considered as Secondary Targets (ST) since no direct interaction was found at *in-silico* level.

1 $$TS=\{\begin{array}{c}(\,+\,LM)(\,+\,PMR)\to \,PT\\ \,(\,+\,LM)(\,-\,PMR)\to \,ST\,\end{array}$$

where, TS is Target Screening, LM represents Literature Mining, PMR denotes PharmMapper Results, PT stands for Primary Targets and ST for Secondary Targets.

### Gene Ontology and Co-Expression Network

To capture comprehensive view of how targets form signaling cascade to inhibit or enhance the disease response and their role in various domains like biological process, molecular function and cellular component; we performed gene ontology analysis. The analysis also gave an idea about the inter-connecting component in which biomarker association with neighboring genes can be identified. Besides, BLAST2GO software was used to identify various interactions between predicted targets on the basis of node score.

2 $$score(g)=\sum _{{g}_{\alpha }\in desc(g)}gp({g}_{\alpha }).{\alpha }^{dist(g,{g}_{\alpha })}$$

where

- *desc*(*g*) represents all the descendant terms for a given GO term *g*
- *dist*(*g*, *ga*) represents the number of edges between the GO term *g* and the GO term *ga*
- *g* represents the element of GO, where GO is the whole set of all GO terms
- *gp*(*g*) represents the number of gene outcomes given to a given GO term *g*

Score is calculated in terms of Biological Processes Score (BPS), Molecular Functions Score (MFS), and Cellular Components Score (CCS). Overall Gene Ontology Score (GOS) is represented as:

3 $$GOS=\sum _{k=1}^{n}\,(BPS)+\,\sum _{l=1}^{n}\,(MFS)+\sum _{m=1}^{n}\,(CCS)\,$$

To elaborate the network and to gain comprehensive knowledge about the targets and their associated partners we downloaded the interacting partners from Search Tool for the Retrieval of Interacting Genes/Proteins (STRING) database^39^, which contains information from several sources, like *in-silico* prediction methods, experimental data and scientific literatures. For network construction analysis both parametric (Pearson Correlation Coefficient (PCC)) and non-parametric test (Spearman Correlation Coefficient (SCC)). But, SCC has not shown significant correlation but PCC showed significant correlation and overlapping results with available literature. Following the collection from STRING database, data was weighted, integrated and a confidence score was calculated for all protein interactions using Pearson Correlation Coefficient (PCC) which measures the linear correlation between two variables.

4 $$PCC=\frac{[{M}^{-1}{\sum }_{i=1\,}^{M}{j}_{i}{k}_{i}]-[{M}^{-1}{\sum }_{i=1}^{M}\frac{1}{2}{({j}_{i}+{k}_{i})}^{2}]}{[{M}^{-1}{\sum }_{i=1}^{M}\frac{1}{2}({{j}_{i}}^{2}+{{k}_{i}}^{2})]-[{M}^{-1}{\sum }_{i=1}^{M}\frac{1}{2}{({j}_{i}+{k}_{i})}^{2}]}$$

where *j*_*i*_ and *k*_*i*_ are the degrees of targets at both the ends of the *i*^*th*^ connection, and *M* represents total connections in the network.

Results of the various *in-silico* predictions were inspected from different designated views. Moreover, normalization and categorization based on the association score of co-expressed modules was performed. Module Construction (MC) was performed by combining all calculated scores given in the MC equation.

5 $$MC=\sum (CES)+(GOS)+\,(CLC)+(BWC)+(DON)$$

where, *CES* stands for Co-Expression Score, *CLC* for Clustering Coefficient, *BWC* for Betweenness Centrality, and *DON* for Degree of Nodes. Information of selected parameters is given below:

A co-expression network is an undirected graph, with every node representing a gene and every edge representing the connection between these nodes. In this study, we used an in-house Perl script to calculate gene co-expression; we calculated various scores, assigned weights to each score, and finally generated a combined score. Methodology was adopted from our previous study on miRNA regulatory network analysis^3^.

Gene Ontology Score deals with three components, namely Biological Processes (BPs), Molecular Functions (MFs), and Cellular Components (CCs). BLAST2GO was used to link selected genes to map with the GO database in terms of BPs, MFs, and CCs. The genes that belonged to the same category were clustered. A node score function was defined for all targeted genes. Genes that had the same score were clustered in the same cluster category. Interconnection from one cluster to another cluster was performed on the basis of their respective association based on the node score.

The degree of a node in an undirected graph is the number of connexions or edges a node has with other nodes, and it is defined as *deg*(*i*) = *k*(*i*) = *|N*(*i*)*|* where *N*(*i*) is the number of the neighbours of node *i*. The degree distribution *p*(*k*) reveals the fraction of vertices with degree *k*. DON gives the idea of association of nodes with node of interest.

Clustering Coefficient is the measurement that shows the tendency of a graph to be divided into clusters. A cluster is a subset of vertices that contains lots of edges connecting these vertices to each other. Assuming that *i* is a vertex with degree *deg*(*i*) = *k* in an undirected graph *G* and that there are *e* edges between the *k* neighbors of *i* in *G*, then the *Clustering Coefficient* of *i* in *G* is given by:

6 $${C}_{i}=\frac{2e}{k(k-1)}$$

Thus, *C*_*i*_ measures the ratio of the number of edges between the neighbors of *i* to the total possible number of such edges, which is *k*(*k* − 1)*/*2. It takes values as 0 ≤ *C*_*i*_ ≤ 1.

Betweenness Centrality shows that nodes which are intermediate between neighbors rank higher. Without these nodes, there would be no way for two neighbors to communicate with each other. Thus, betweenness centrality shows important nodes that lie on a high proportion of paths between other nodes in the network. For distinct nodes *i*, *j*, *w* ∈ *V*(*G*), let *σ*_*ij*_ be the total number of shortest paths between *i* and *j* and *σ*_*ij*_(*w*) be the number of shortest paths from *i* to *j* that pass through *w*. Moreover, for *w* ∈ *V*(*G*), let *V* (*i*) denote the set of all ordered pairs, (*i*, *j*) in *V*(*G*) × *V*(*G*) such that *i*, *j*, *w* are all distinct. Then, the Betweenness Centrality is calculated as:

7 $${C}_{b}(w)=\sum _{(ij)\in V(w)}\frac{{\sigma }_{ij}(w)}{{\sigma }_{ij}}$$

### Pathway Mapping of Co-Expressed Modules

After identifying co-expressed gene modules, a mapping of associated partners with designated pathway was performed by manual literature survey followed by constructing static map using KEGG, REACTOME and Pathway Interaction Database (PID) as a reference pathway maps, to aid proper understanding of molecular mechanism of action and target implication^40–42^.

### Patch Dock Analysis

Also, to understand the inhibitory role of selected targets with small molecules (P-I and P-II), molecular docking was performed based on shape complimentary principles using PatchDock web server^33^; as we are interested to observe variations in target binding energy of picroside derivates with already known drug targets.

## Electronic supplementary material

Supplementary File 1

Supplementary Dataset 1
