# Supplementary material for: An integrative approach to develop computational pipeline for drug-target interaction network analysis
Source: Sci Rep. 2018 Jul 6;8:10238. doi: 10.1038/s41598-018-28577-6 (PMC6035197; doi:10.1038/s41598-018-28577-6)
Supplement: Supplementary file 1 [file 41598_2018_28577_MOESM1_ESM.pdf]

An integrative approach to develop computational pipeline for drug-target interaction network analysis

Ankush Bansal, Pulkit Anupam Srivastava, Tiratha Raj Singh*

Department of Biotechnology and Bioinformatics, Jaypee University of Information Technology, Waknaghat- 173234, Solan (HP), India

***Corresponding author:**

Dr. Tiratha Raj Singh

Email: tiratharaj@gmail.com

Scoping Document : Picrorhiza kurroa [Picrosides]

Title: Picroside II Attenuates CCI-Induced Neuropathic Pain in Rats by Inhibiting Spinal Reactive Astrocyte-Mediated Neuroinflammation Through the NF-κB Pathway.

PMID: 29671236

Publication Date: 2018 May

Gist: Reactive astrocyte-mediated neuroinflammatory responses in the spinal dorsal horn have been reported to play a pivotal role in pathological pain. Chronic constriction injury (CCI) enhances the activation of nuclear factor kappa B (NF-κB), which is involved in neuropathic pain (NP). Picroside II (PII), a major active component of Picrorhiza scrophulariiflora, has been investigated for its anti-oxidative, anti-inflammatory, and anti-apoptotic activities. Here, we explored the analgesic effects of PII on a model of CCI-induced NP and investigated the levels of the GFAP protein and the mRNA and protein levels of pro-inflammatory cytokines in the spinal cord, including interleukin-1β (IL-1β), interleukin-6 (IL-6) and tumor necrosis factor-α (TNF-α). CCI significantly induced mechanical allodynia and thermal hyperalgesia. Intraperitoneal administration of PII remarkably reversed the CCI-induced mechanical allodynia and thermal hyperalgesia and reduced the mRNA and protein levels of IL-1β, IL-6, and TNF-α in the spinal cord. Additionally, according to the in vitro data, the PII treatment inhibited LPS-induced increases in the mRNA and protein levels of IL-1β, IL-6, and TNF-α and suppressed the NF-κB pathway by inhibiting the phosphorylation of NF-κB/p65 and the degradation of inhibitor of NF-κB (IκB) in astrocytes without toxicity to astrocytes. Overall, the analgesic effect of PII correlated with the inhibition of spinal reactive astrocyte-mediated neuroinflammation through the NF-κB pathway in rats with NP.

Title: Effect of picroside II on the expression of mitochondrial VDAC1 after cerebral ischemia/reperfusion in rats.

PMID: 29343040

Publication Date: 2018 Jan

Gist: Picroside II could attenuate cerebral I/R injury by down-regulating the expression of VDAC1 and inhibiting the EndoG release from mitochondria into cytoplasm.

Title: Picroside II Exerts a Neuroprotective Effect by Inhibiting mPTP Permeability and EndoG Release after Cerebral Ischemia/Reperfusion Injury in Rats.

PMID: 29256102

Publication Date: 2018 Jan

Gist: Mitochondrial membrane permeability is closely related to cerebral ischemia/reperfusion (I/R) injury. This paper explored the neuroprotective effect of picroside II (Picr), which inhibits

the permeability of mitochondrial permeability transition pore (mPTP) and endonuclease G (EndoG) release from mitochondria into cytoplasm after cerebral I/R in rats. After 2 h of cerebral ischemia and 24 h of reperfusion in rats with different intervention measures, the neurobehavioral function, infarction volume, and reactive oxygen species (ROS) content in brain tissues were observed by modified neurological severity scale (mNSS), triphenyl tetrazolium chloride (TTC) staining, and enzyme-linked immunosorbent assay, respectively. The permeability of mPTP was assayed using spectrophotometry. The morphology and apoptotic cells of brain tissues were observed by hematoxylin-eosin staining and terminal deoxynucleotidyl transferase dUTP nick end labeling assay, respectively. The expressions of EndoG and voltage-dependent anion channel 1 (VDAC1) were determined by immunohistochemical assay and western blot. The Picr group exhibited clear decreases in mNSS scores, ROS content, number of apoptotic cells, mPTP permeability and expression of VDAC1, and EndoG in cytoplasm and nuclei, and the morphology of brain tissue was improved as compared with the model group ($P < 0.05$). Picr could attenuate cerebral I/R injury by downregulating the expression of VDAC1 and decreasing the permeability of mPTP, thereby inhibiting EndoG release from mitochondria into cytoplasm.

Title: Picroside II attenuates fatty acid accumulation in HepG2 cells via modulation of fatty acid uptake and synthesis.

PMID: 27328362

Publication Date: 2018 Mar

Gist: Picroside II pretreatment inhibited FFAs-induced lipid accumulation by attenuating the expression of fatty acid transport protein 5, sterol regulatory element binding protein 1 and stearoyl CoA desaturase. Preatreatment with picroside II was also found to decrease the expression of forkhead box protein O1 and phosphoenolpyruvate carboxykinase.

Title: *Picrorhiza kurroa* Enhances *β*-Cell Mass Proliferation and Insulin Secretion in Streptozotocin Evoked *β*-Cell Damage in Rats.

PMID: 28878669

Publication Date: 2017 Aug

Gist: Autoimmune destruction of insulin producing pancreatic β-cells leads to insulin insufficiency and hyperglycemia in type 1 diabetes mellitus. Regeneration of β-cells is one of the proposed treatment for type 1 diabetes and insulin insufficiency. *Picrorhiza kurroa* is a medicinal herb and is traditionally being used for the treatment of various diseases. Previous studies reported the hypoglycemic potential of *P. kurroa*. However, its potential role in β-cell induction in insulin secretion have not been fully investigated. Here, we characterized the hydro alcoholic extract of *P. kurroa* rhizome (PKRE) and further studied its β-cell regeneration and induction of insulin secretion potential in streptozotocin (STZ) induced diabetic rats as well as in insulin producing Rin5f cells. ^{1}H-NMR revealed the presence of more than thirty metabolites including picroside I and II in PKRE. Further, we found that PKRE treatment (100 and 200 mg/kg dose for 30 days) significantly ($p \leq 0.05$) protected the pancreatic β-cells against streptozotocin (STZ) evoked damage and inhibited the glucagon receptor expression (Gcgr) in

hepatic and renal tissues. It significantly ($p \leq 0.05$) enhanced the insulin expression and aids in proliferation of insulin producing Rin5f cells with elevated insulin secretion. Furthermore it significantly ($p \leq 0.05$) increased insulin mediated glucose uptake in 3T3L1 and L6 cells. On the contrary, in diabetic rats, PKRE significantly ($p \leq 0.05$) decreased high blood glucose and restored the normal levels of serum biochemicals. Altogether, our results showed that PKRE displayed β-cell regeneration with enhanced insulin production and antihyperglycemic effects. PKRE also improves hepatic and renal functions against oxidative damage.

Title: Effect of picroside II on hind limb ischemia reperfusion injury in rats.

PMID: 28721011

Publication Date: 2017 Jun

Gist: The results of this study demonstrated that picroside II plays a critical role to prevent I/R injury. Even though our results were found to be satisfactory

---

Title: Picroside II Shows Protective Functions for Severe Acute Pancreatitis in Rats by Preventing NF-*κ*B-Dependent Autophagy.

PMID: 28713490

Publication Date: 2017 Jun

Gist: Picroside II, from the herb *Picrorhiza scrophulariiflora Pennell*, has antioxidant and anti-inflammatory activities. However, its function on severe acute pancreatitis (SAP) and molecular mechanism remains unknown. The effects of picroside II on the SAP induced by cerulean were investigated. SAP rats were treated with picroside II (25 mg/kg). The severity of SAP was evaluated by using biochemical and histological analyses. Pancreatic cancer cell PANC-1 was transfected with ptfLC3 (an indicator of autophagic activity), pcDNA3.1-NF-*κ*B (nuclear factor kappa B), and pTZU6+1-NF-*κ*B-shRNA and then treated with picroside II. Relative molecules related with NF-*κ*B-dependent autophagy were detected by using Western blot. Autophagic activities were observed by phase-contrast and fluorescent microscopes. Acetylated LC3 was detected by immunoprecipitation. The results showed that picroside II treatment reduced the level of ALT, AST, NF-*κ*B, IL-1*β*, IL-6, TNF-*α*, and SIRT1 (NAD^+-dependent deacetylase) and increased the level of SOD and GSH. The autophagic activity was reduced when NF-*κ*B was silenced, and the levels of TNF-*α* and SIRT1 were reduced. In contrast, the overexpression of NF-*κ*B increased autophagic activity and the level of TNF-*α*, which activated SIRT1. SIRT1 deacetylated LC3 and increased autophagic activities. Picroside II ameliorates SAP by improving antioxidant and anti-inflammtory activities of SAP models via NF-*κ*B-dependent autophagy.

Title: Picroside II Inhibits RANKL-Mediated Osteoclastogenesis by Attenuating the NF-κB and MAPKs Signaling Pathway In Vitro and Prevents Bone Loss in Lipopolysaccharide Treatment Mice.

PMID: 28464271

Publication Date: 2017 Dec

Gist: Picroside II, one of the major components isolated from the seed of natural plant picrorhiza, is widely used in traditional Chinese medicine. The present study was performed to define effects of picroside II on nuclear factor-kappaB ligand (RANKL)-stimulated osteoclast differentiation in vitro and on lipopolysaccharide (LPS)-induced bone loss in vivo. The bone marrow cells (BMMs) were harvested and induced with RANKL followed by treatment with picroside II at several doses, and the differentiation of osteoclasts from these cells was evaluated by tartrate-resistant acid phosphatase (TRAP) staining and resorption pit formation assay. The effects of picroside II on osteoclastogenesis were studied by examining RANKL-induced osteoclast F-actin ring formation and osteoclast bone resorption. Moreover, we explored the mechanisms of these downregulation effects by performed Western blotting and quantitative RT-PCR examination. Results demonstrated picroside II strongly inhibited RANKL-induced osteoclast formation when added during the early stage of BMMs cultures, suggesting that it acts on osteoclast precursors to inhibit RANKL/RANK signaling. Moreover, picroside II markedly decreased the phosphorylation of p38, ERK, JNK, p65, and I-κB degradation, and significantly suppressed c-Fos and nuclear factor of activated T-cells cytoplasmic 1 (NFATc1), both the key transcription factors during osteoclastogenesis. Furthermore, in vivo studies verified the bone protection effects of picroside II. These results collectively suggested that picroside II acted as an anti-resorption agent by blocking osteoclast activation.

Title: Picroside II protects the blood-brain barrier by inhibiting the oxidative signaling pathway in cerebral ischemia-reperfusion injury.

PMID: 28388666

Publication Date: 2017 Apr

Gist: A higher neurological score, bigger cortex infarction, more damaged neuron structure and injured BBB, increased content of ROS and activity of NADPH oxidase, higher protein levels of Nox2, Rac-1, ROCK, MLCK and MMP-2 and lower levels of claudin-5 were observed in the model group. In the picroside group, the neurological score, neuronal damage, BBB injury, ROS content and NADPH oxidase activity were reduced ($P<0.05$), and the protein levels of Rac-1, Nox2, ROCK, MLCK and MMP-2 were down-regulated ($P<0.05$), while the expression of claudin-5 was up-regulated ($P<0.05$). Picroside II could protect the nervous system possibly through reducing the content of ROS by down-regulating the expression of Rac-1 and Nox2 and could protect the BBB through reducing the expression of ROCK, MLCK, and MMP-2, while enhancing the expression of claudin-5.

Title: Picroside II Protects Rat Lung and A549 Cell Against LPS-Induced Inflammation by the NF-κB Pathway.

PMID: 28161732

Publication Date: 2017 Jun

Gist: Picroside II is the main active ingredient in the root department of Chinese medicine Picrorhiza scrophulariiflora which has been proved to have beneficial effects on health, such as

ameliorating the cerebral ischemia and protecting the liver. However, its effects on acute lung injury remain unclear. The purpose of the study was to evaluate the effects of picroside II on acute lung injury in mice and the inflammation in A549 cells which are lipopolysaccharide (LPS) induced. We evaluated the levels of tumor necrosis factor-α (TNF-α), interleukin-1 (IL-1β), and interleukin-6 (IL-6) in vivo and vitro by enzyme-linked immunosorbent assay (ELISA) and quantitative polymerase chain reaction (qRT-PCR). Results showed picroside II significantly decreased the concentrations of TNF-α, IL-1β, and IL-6 in cells and mice. In addition, Western blot and immunofluorescence analysis indicated that picroside II suppressed the activation of p65 NF-κB signaling pathway compared with LPS stimulation. In the acute lung injury model of mice, after picroside II treatment, the pathologic changes of lung tissues had been alleviated and lung wet/dry weight ratio was decreased. In summary, picroside II showed the promising effects of anti-inflammation in cells and animals.

Title: Picroside II protects myocardium from ischemia/reperfusion-induced injury through inhibition of the inflammatory response.

PMID: 28105084

Publication Date: 2016 Dec

Gist: The inflammatory response is important in the pathogenesis of myocardial ischemia/reperfusion (I/R) injury. Picroside II, the primary active constituent of Picrorhizae, has been reported to protect the myocardium from I/R-induced injury, however, the exact mechanism underlying these protective effects remains unclear. The aim of the present study was to investigate the mechanism underlying the protective effects of picroside II on I/R-induced myocardial injury. Adult male Sprague-Dawley rats underwent 1 h left coronary artery occlusion followed by 3 h reperfusion. Picroside II was administered (10 mg/kg) via the tail vein 30 min prior to left coronary artery occlusion. The results revealed that pretreatment of picroside II could significantly alleviate I/R-induced myocardial injury concomitantly with a decrease in inflammatory factor production. In addition, picroside II was also able to decrease high mobility group box 1 (HMGB1) expression, and release and downregulate the expression of the receptor for advanced glycation end products (RAGE), toll-like receptor (TLR)-2 and TLR-4. Furthermore, picroside II was able to inhibit nuclear factor-κB (NF-κB) activation. The results indicated that the protective effect of picroside II on I/R-induced myocardial injury was associated, at least partly, with inhibition of the inflammatory response by suppressing the HMGB1-RAGE/TLR-2/TLR-4-NF-κB signaling pathway.

Title: Neuroprotective effect of picroside II in brain injury in mice.

PMID: 28078024

Publication Date: 2016 Dec

Gist: Various types of brain injury which led to the damage of brain tissue structure and neurological dysfunction continues to be the major causes of disability and mortality. Picroside II (PII) possesses a wide range of pharmacological effects and has been proved to ameliorate ischemia and reperfusion injury of kidney and brain. However, critical questions remain about

other brain injuries. We investigated the protective effect of PII in four well-characterized murine models of brain injury. Models showed a subsequent regional inflammatory response and oxidative stress in common, which might be improved by the administration of PII (20 mg/kg). Meanwhile, a series of morphological and histological analyses for reinforcement was performed. In traumatic, ischemic and infectious induced injuries, it was observed that the survival rate, apoptosis related proteins, Caspase-3, and the expression of acute inflammatory cytokines (IL-1β, IL-6 and TNF-α) were significantly alleviated after PII injection, but PII treatment alone showed no effect on them as well. The western blot results indicated that TLR4 and NF-κB were clearly downregulated with PII administration. In conclusion, our results suggested that PII with a recommended concentration of 20 mg/kg could provide neuroprotective effects against multi-cerebral injuries in mice by suppressing the over-reactive inflammatory responses and oxidative stress and attenuating the damage of brain tissue for further neurological recovery.

Title: Picroside II protects against sepsis via suppressing inflammation in mice.

PMID: 28078023

Publication Date: 2016 Dec

Gist: Picroside II, an iridoid compound extracted from Picrorhiza, exhibits anti-inflammatory and anti-apoptotic activities. We explored the protective effects and mechanisms of picroside II in a mouse model of sepsis induced by cecal ligation and puncture (CLP), using three groups of mice: Group A (sham), Group B (CLP+NS) and Group C (CLP+20 mg/kg picroside II). The mortality in mice with sepsis was decreased by the administration of picroside II, and lung injury was alleviated simultaneously. Picroside II treatment enhanced bacterial clearance in septic mice. Further, picroside II treatment alleviated the inflammatory response in sepsis and enhanced immune function by inhibiting the activation of NLRP3 inflammasome and NF-κB pathways. Picroside II may represent an anti-inflammatory drug candidate, providing novel insight into the treatment of sepsis.

Title: Picroside II Exerts a Neuroprotective Effect by Inhibiting the Mitochondria Cytochrome C Signal Pathway Following Ischemia Reperfusion Injury in Rats.

PMID: 28054226

Publication Date: 2017 Feb

Gist: Stroke is a common neurodegenerative disease in the wide world, and mitochondrial defects underlie the pathogenesis of ischemia, especially during reperfusion. Picroside II, the principal active component of Picrorhiza, is a traditional Chinese medicine. Our previous study demonstrated that the best therapeutic dose and time window were injection of picroside II at a dose of 10-20 mg/kg body weight following cerebral ischemia by 1.5-2.0 h. In this paper, the neuroprotective effect and the mechanism of picroside II were investigated, as well as its involvement in antioxidant and mitochondria cytochrome C (CytC) signal pathway following ischemia reperfusion (I/R) injury in rats. After 24 h of cerebral I/R, the neurobehavioral function

was measured by modified neurological severity score test; the content of reactive oxygen species in brain tissue was measured by enzyme-linked immunosorbent assay; the cerebral infarction volume was detected by TTC staining; the morphology of brain tissue was observed by hematoxylin-eosin; the apoptotic cells were counted by terminal deoxynucleotidyl transferase dUTP nick end labeling assay; the ultrastructure of the cortical brain tissues was observation by transmission electron microscopy; the expressions of CytC and Caspase-3 were determined by immunohistochemical assay and Western blot. The results indicated that picroside II could scavenge ROS contents, decrease the cerebral infarction volume and apoptotic cells, protect the structure of mitochondria, down-regulate the expression of CytC and Caspase-3 in cerebral I/R rats. It can be concluded that picroside II exerts a neuroprotective effect by inhibiting the mitochondria CytC signal pathway following ischemia reperfusion injury in rats.

Title: Picroside II Attenuates Airway Inflammation by Downregulating the Transcription Factor GATA3 and Th2-Related Cytokines in a Mouse Model of HDM-Induced Allergic Asthma.

PMID: 27870920

Publication Date: 2016 Nov

Gist: Picroside II isolated from Pseudolysimachion rotundum var. subintegrum has been used as traditional medicine to treat inflammatory diseases. In this study, we assessed whether picroside II has inhibitory effects on airway inflammation in a mouse model of house dust mite (HDM)-induced asthma. In the HDM-induced asthmatic model, picroside II significantly reduced inflammatory cell counts in the bronchoalveolar lavage fluid (BALF), the levels of total immunoglobulin (Ig) E and HDM-specific IgE and IgG1 in serum, airway inflammation, and mucus hypersecretion in the lung tissues. ELISA analysis showed that picroside II down-regulated the levels of Th2-related cytokines (including IL-4, IL-5, and IL-13) and asthma-related mediators, but it up-regulated Th1-related cytokine, IFNγ in BALF. Picroside II also inhibited the expression of Th2 type cytokine genes and the transcription factor GATA3 in the lung tissues of HDM-induced mice. Finally, we demonstrated that picroside II significantly decreased the expression of GATA3 and Th2 cytokines in developing Th2 cells, consistent with in vivo results. Taken together, these results indicate that picroside II has protective effects on allergic asthma by reducing GATA3 expression and Th2 cytokine bias.

Title: Characterization of picroside II metabolites in rats by ultra-high-performance liquid chromatography combined with electrospray ionization quadrupole time-of-flight tandem mass spectrometry.

PMID: 27328362

Publication Date: 2016 Jun 7

Gist: Picroside II reported to have hepatoprotective, neuroprotective, and antioxidant effects. In the first pathway,picroside II is deglycosylated to generate aglycone, which is isomerized to a dialdehyde-type intermediate. A series of metabolic reactions, including glucuronidation, subsequently occurs. In the second pathway, picroside II is subjected to ester bond hydrolysis to form vanillic acid, which is further subjected to sulfate conjugation, glycine conjugation, glucuronidation, and demethylation. In the third pathway, picroside II is directly conjugated with

glucuronic acid to yield a predominant metabolite (M01) in plasma. In the fourth pathway, picroside II is directly conjugated with sulfate.

Title: Effect of Picroside II on ERK1/2 Signal Pathway in Cerebral lschemic Injury Rats.

PMID: 27323616

Publication Date: 2016 Apr

Gist: The neuroprotective effect and mechanism of picroside II on extracellular regulated protein kinases1/2 (ERK1/2) signal transduction pathway in cerebral ischemia injuryrats. Activating ERK12 pathway could mediate apoptosis and inflammatory reactions of neurons after cerebral ischemia injury.Picroside II could protect the nerve system possibly through reducing activation of ERKI2 pathway, inhibiting apoptosis of neurons and inflammation reaction.

Title: In vitro - In vivo metabolism and pharmacokinetics of picroside I and II using LC-ESI-MS method.

PMID: 27234049

Publication Date: 2016 Jul 25

Gist: The rhizomes of P. kurroa have been traditionally used to treat worms, constipation, low fever, scorpion sting, asthma and ailments affecting the liver. In metabolic study, eight metabolites of picroside I and six metabolites of picroside II were identified in vitro, out of which four metabolites for each picroside I and picroside II were identified in vivo.

Title: Effect of picroside II on erythrocyte deformability and lipid peroxidation in rats subjected to hind limb ischemia reperfusion injury.

PMID: 27041996

Publication Date: 2016 Mar 1

Gist: Ischemia reperfusion injury (I/R) in hind limb is a frequent and important clinical phenomenon. Many structural and functional damages are observed in cells and tissues in these kinds of injuries. In this study, aimed to evaluate the effect of picroside II on lipid peroxidation and erythrocyte deformability during I/R in rats. These results support that deformability of erythrocytes is decreased in I/R injury and picroside II plays a critical role to prevent these alterations. Further experimental and clinical studies are needed to evaluate and clarify the molecular mechanisms of action and clinical importance of these findings.

Title: Neutrophilic Lung Inflammation Suppressed by Picroside II Is Associated with TGF-β Signaling.

PMID: 26617662

Publication Date: 2015 Nov 5

Gist: The rhizome of Picrorhiza scrophulariiflora used in a traditional herbal medicine in Asian countries has been shown to have anti-inflammatory function, and picroside II (PIC II) is known as a major constituent in the plant. Here, examined whether PIC II has an anti-inflammatory activity, which is applicable for treating ALI. We found that although it is not significantly effective in suppressing proinflammatory factor NF-κB or in activating anti-inflammatory factor Nrf2, PIC II induced the phosphorylation of Smad 2, with concomitant increase of luciferase activity from SBE luciferase reporter in RAW 264.7 cells. H&E staining of lung, differential counting of cells in bronchoalveolar lavage fluid, and semiquantitative RT-PCR analyses of lung tissues show that an intratracheal (i.t.) spraying of PIC II suppressed neutrophilic inflammation and the expression of proinflammatory cytokine genes in the lung, which were elicited by an i.t. LPS instillation to the lung. In addition, PIC II treatment increased the phosphorylation of Smad 2 in the lung tissue. Together, our results suggest that PIC II plays a role as an anti-inflammatory constituent in P. scrophulariiflora, whose activity is associated at least in part with TGF-β signaling

---

Title: Picroside II Inhibits the MEK-ERK1/2-COX2 Signal Pathway to Prevent Cerebral Ischemic Injury in Rats.

PMID: 26240040
Publication Date: 2015 Aug 4

Gist: The objective of this study is to explore the neuroprotective effect and mechanism of picroside II on ERK1/2-COX2 signal transduction pathway after cerebral ischemic injury in rats. In the picroside and U0126 groups, the neurological behavioral function was improved, and

the number of apoptotic cells and the expression of pMEK1/2, pERK1/2, and COX2 decreased significantly when compared to the model group. In the LPS withpicroside group, at ischemia 6 h neuron damage was extensive, and pMEK1/2, pERK1/2, and COX2 expression were much higher than in the model group. But at ischemia 12 and 24 h, the expression of pMEK1/2, pERK1/2, and COX2 decreased slightly, and the neurobehavioral function also improved slightly. In LPS group, neuron damage was extensive, pMEK1/2, pERK1/2, and COX2 expression was still at a high level, and COX2 mRNA peak arrived at ischemic 12 h. Picroside II downregulates COX2 expression after MCAO by inhibiting MEK-ERK1/2 in rats to protect neurons from apoptosis and inflammation.

Title: Picroside II as a Novel Inhibitor of Apoptosis After Cerebral Ischemia in Rats.

PMID: 26181788

Publication Date: 2015 Aug

Gist: NA

Title: Picroside II has a neuroprotective effect by inhibiting ERK1/2 activation after cerebral ischemic injury in rats.

PMID: 26175147

Publication Date: 2015 Jul 14

Gist: In the study, we explored the neuroprotective effect and underlying mechanism of picroside II involving the ERK1/2 signal pathway after cerebral ischemia injury in rats. The results suggests that activation of ERK1/2 in cerebral ischaemia induces neuronal apoptosis and picroside II may reduce neuronal apoptosis to confer protection against cerebral ischemic injury by inhibiting ERK1/2 activation.

Title: A pre-clinical pharmacokinetic study in rats of three naturally occurring iridoid glycosides, Picroside-I, II and III, using a validated simultaneous HPLC-MS/MS assay.

PMID: 25984965

Publication Date: 2015 Jul 1

Gist: Picroside-I, II, and III were all eliminated rapidly with large volume of distribution. Among the three glycosides, Picroside-II showed the highest liver uptake, and only Picroside-I and II were found to get across the blood brain barrier (BBB). These results were consistent with their hepatoprotective or neuroprotective effects reported clinically.

Title: Picroside II Inhibits Neuronal Apoptosis and Improves the Morphology and Structure of Brain Tissue following Cerebral Ischemic Injury in Rats.

PMID: 25927985

Publication Date: 2015 Apr 30

Gist: This paper aimed to explore the protective effects of picroside II against the neuronal apoptosis and changes in morphology and structure that follow cerebral ischemic injury in rats. These results suggest that picroside II reduced apoptosis and improved the morphology and ultrastructure of the neurons and the BBB and that these effects resulted in the recovery of the neurobehavioral function of rats with cerebral ischemia.

Title: Picroside II protects rat kidney against ischemia/reperfusion-induced oxidative stress and inflammation by the TLR4/NF-κB pathway.

PMID: 25780418

Publication Date: 2015 Apr

Gist: Picroside II possesses a wide range of pharmacological effects and has been demonstrated to ameliorate cerebral ischemia and reperfusion (I/R) injury. However, its effects on renal I/R injury remain unclear. In the present study, the role of picroside II in attenuating oxidative stress and the inflammatory response in a rat model of renal I/R injury was investigated. It was observed that renal function was significantly improved by treatment with picroside II. Morphological analysis indicated that picroside II clearly reduced tissue damage and the

expression of TLR4 and NF-κB. Reverse transcription-quantitative polymerase chain reaction demonstrated that picroside II inhibited the increase of tumor necrosis factor (TNF)-α, interleukin (IL)-1β and intercellular adhesion molecule (ICAM)-1 expression induced by I/R injury. Western blot analysis indicated that the expression levels of TLR4 and NF-κB were significantly downregulated in the picroside II group compared with those in the I/R group. These results indicate that picroside II treatment suppressed the TLR4/NF-κB signaling pathway, protecting renal tissue against I/R-induced oxidative stress and inflammatory response.

Title: Effect of picroside II on apoptosis induced by renal ischemia/reperfusion injury in rats.

PMID: 25667634

Publication Date: 2015 Mar

Gist: Picroside II possesses a wide range of pharmacological effects, including anti-apoptosis effects. In the present study, the ability of picroside II to attenuate apoptosis in a rat model of renal I/R injury was investigated. It was observed that renal function was significantly improved by the treatment with picroside II. Morphological analysis indicated that picroside II markedly reduced tissue damage and the expression of cleaved caspase-3. Reverse transcription-quantitative polymerase chain reaction and western blotting revealed that the expression levels of Bax and poly(ADP-ribose) polymerase-1 (PARP-1) were upregulated in the I/R group, whereas those of Bcl-2 were downregulated. However, the treatment with picroside II inhibited these changes induced by renal I/R injury. In conclusion, picroside II has potent anti-apoptotic activity against renal I/R injury.

Title: Picroside II inhibits hypoxia/reoxygenation-induced cardiomyocyte apoptosis by ameliorating mitochondrial function through a mechanism involving a decrease in reactive oxygen species production.

PMID: 25421707

Publication Date: 2015 Feb

Gist: Reactive oxygen species (ROS)-induced mitochondrial dysfunction plays an important role in cardiomyocyte apoptosis during myocardial ischemia/reperfusion (I/R) injury. Picroside Ⅱ, isolated from Picrorhiza scrophulariiflora Pennell (Scrophulariaceae), has been reported to protect cardiomyocytes from hypoxia/reoxygenation (H/R)-induced apoptosis, but the exact mechanism is not fully clear. The aim of the present study was to explore the protective effects of picroside Ⅱ on H/R-induced cardiomyocyte apoptosis and the underlying mechanism. In the H9c2 rat cardiomyocyte cell line, picroside Ⅱ (100 μg/ml) was added for 48 h prior to H/R. The results showed that picroside Ⅱ markedly inhibited H/R-induced cardiomyocyte apoptosis. In addition, picroside Ⅱ was also able to decrease the opening degree of mitochondrial permeability transition pore (mPTP), increase the mitochondrial membrane potential, inhibit cytochrome c release from mitochondria to cytosol and downregulate caspase-3 expression and activity concomitantly with the decreased ROS production. These results suggested that

picroside II inhibited H/R-induced cardiomyocyte apoptosis by ameliorating mitochondrial function through a mechanism involving a decrease in ROS production.

Title: Picroside II decreases the development of fibrosis induced by ischemia/reperfusion injury in rats.

PMID: 25246345

Publication Date: 2014 Oct

Gist: Investigated the role of the Picroside II, the main active constituents of the extract of picrorrhiza scrophulariiflora roots, in attenuating renal fibrosis in a renal ischemia and reperfusion injury model. We induced ischemia and reperfusion injury in kidneys treated with or without Picroside II. We observed that inflammation and tissue fibrosis were increased in ischemia and reperfusion injury group compared to Picroside II group, however, these changes were significantly decreased by the treatment with Picroside II. We concluded that Picroside II can protect the ischemic kidney against renal fibrosis and its mechanism may be through the inhibition of the long term inflammation.

Title: Protective effect of picroside II on myocardial ischemia reperfusion injury in rats.

PMID: 24868147

Publication Date: 2014 May 14

Gist: The aim of this study was to determine the effect of picroside II on myocardial ischemia reperfusion injury in rats and to explore its underlying mechanism. Different doses of picroside II (1 μM, 10 μM, and 100 μM) were given 20 minutes before ischemia. Phosphoinositide 3-kinase inhibitor (wortmannin) and nitric oxide synthase (NOS) inhibitor (L-N(G)-nitroarginine methyl ester) were given 10 minutes before picroside II treatment. The cardiac function, myocardial infarct size, apoptosis, myocardial nitric oxide content, the expressions of Bcl-2 and Bax, and the activation of the phosphoinositide 3-kinase/Akt/endothelial NOS pathway were evaluated. Treatment with 10 μM and 100 μM picroside II significantly improved postischemic myocardial function, reduced myocardial infarct size, inhibited apoptosis, increased myocardial NO content, upregulated Bcl-2, downregulated Bax, and increased the phosphorylation of Akt and endothelial NOS, but cardioprotection was not shown in the 1 μM picroside II treatment group and was abrogated by wortmannin and L-N(G)-nitroarginine methyl ester. Furthermore, cardioprotection in the 100 μM picroside II treatment group was superior to that in the 10 μM picroside II treatment group. In conclusion, the data reveals that picroside II has a significant protective effect on myocardial ischemia reperfusion injury in a dose-dependent manner, which was mediated by upregulating the phosphoinositide 3-kinase/Akt/endothelial NOS pathway to increase nitric oxide production and regulating the expressions of Bcl-2 and Bax to inhibit apoptosis.

Title: The neuroprotective effect of picroside II via regulating the expression of myelin basic protein after cerebral ischemia injury in rats.

PMID: 24524292

Publication Date: 2014 Feb 14

Gist: The protective effect of picroside II was presented by increasing the expression of MBP and decreasing demyelination after cerebral ischemic injury. The best therapeutic time window and dose was (1) ischemia 2.0 h with picroside II 10 mg/kg body weight according to the results of FGS, IHC and WB; (2) ischemia 1.5 h with picroside II 20 mg/kg according to the analysis of RT-PCR.

Title: Comparative pharmacokinetic profiles of picrosides I and II from kutkin, Picrorhiza kurroa extract and its formulation in rats.

PMID: 23333583

Publication Date: 2013 Mar

Gist: Picrosides I and II are the active chemical constituents, present in the roots and rhizomes of Picrorhiza kurroa Royle (family: Scrophulariaceae). The plant is ethnomedically claimed for the treatment of liver and upper respiratory tract infection, fever, dyspepsia and scorpion sting. This study attempts to determine the in vivo pharmacokinetic profile of picrosides I and II in rats after oral administration of three different preparations namely, kutkin (a mixture of picrosides I and II), P. kurroa extract and Picrolax® capsule (marketed formulation).

Title: The protective effect of picroside II against hypoxia/reoxygenation injury in neonatal rat cardiomyocytes.

PMID: 22880952

Publication Date: 2012 Oct

Gist: Analyzed the effective half-maximal concentration for protection from the dose-response curves and obtained the concentration of 50 μg/mL as EC(50). Pretreated cardiomyocytes with picroside II (50-200 μg/mL), prior to H/R exposure, inhibited LDH activity in culture media and increased cell viability in a dose-dependent manner. This protective effect was accompanied by significantly increasing reduced GSH contents and the activities of SOD and GSH-Px and attenuating MDA and GSSG contents in response to H/R injury. Furthermore, treatment with picroside II also inhibited ROS production and calcium accumulation in cardiomyocytes. The present study demonstrates that picroside II protects cardiomyocytes against oxidative-stress injury induced by H/R through reduction of ROS production and calcium accumulation and enhancement of the activity of antioxidant defense.

Title: Picroside II protects cardiomyocytes from hypoxia/reoxygenation-induced apoptosis by activating the PI3K/Akt and CREB pathways.

PMID: 22581361

Publication Date: 2012 Aug

Gist: Picroside II, an iridoid glucoside found in the root of Picrorhiza scrophulariiflora Pennell (Scrophulariaceae), has been demonstrated to reduce apoptosis in neuronal cells and other cell types. However, whether picroside II has a protective effect against cardiomyocyte apoptosis is poorly understood. In the present study, we investigated the cardioprotective role of picroside II and the underlying mechanisms in hypoxia/reoxygenation-induced cardiomyocyte apoptosis. The pretreatment with picroside II markedly attenuated hypoxia/reoxygenation-induced cell damage dose-dependently, which was evident by the increased cell viability and the corresponding decrease in lactate dehydrogenase release (LDH). The pretreatment with picroside II inhibited apoptosis confirmed by Annexin V-FITC staining, Hoechst 33258 nuclear staining and by assessment of caspase-3 activity. In addition, we found that picroside II not only increased the expression of Bcl-2, while decreasing Bax expression, but also augmented Akt and cAMP response element-binding protein (CREB) phosphorylation and ultimately inhibited hypoxia/reoxygenation-induced apoptosis. Furthermore, the protective effects of picroside II were abrogated by pretreatment of the cells with wortmannin or LY294002, a specific PI3K inhibitor. The present study suggests that picroside II inhibits hypoxia/reoxygenation-induced apoptosis in cardiomyocytes by activating the PI3K/Akt and CREB pathways and modulating expression of Bcl-2 and Bax.

Title: Primary study for the therapeutic dose and time window of picroside II in treating cerebral ischemic injury in rats.

PMID: 2489110

Publication Date: 2012

Gist: The aim of this study was to explore the optimal therapeutic dose and time window of picroside II for treating cerebral ischemic injury in rats according to the orthogonal test.

Title: Assessment of UDP-glucuronosyltransferase catalyzed formation of Picroside II glucuronide in microsomes of different species and recombinant UGTs.

PMID: 21524190

Publication Date: 2011 Jul

Gist: This study compared the hepatic glucuronidation of Picroside II in different species and characterized the glucuronidation activities of human intestinal microsomes (HIMs) and recombinant human UDP-glucuronosyltransferases (UGTs) for Picroside II. The rank order of hepatic microsomal glucuronidation activity of Picroside II was rat > mouse > human > dog. The intrinsic clearance of Picroside II hepatic glucuronidation in rat, mouse and dog was about 10.6-, 6.0- and 2.3-fold of that in human, respectively. Among the 12 recombinant human UGTs, UGT1A7, UGT1A8, UGT1A9 and UGT1A10 catalyzed the glucuronidation. UGT1A10, which are expressed in extrahepatic tissues, showed the highest activity of Picroside II glucuronidation ($K(m) = 45.1$ μM, $V(max) = 831.9$ pmol/min/mg protein). UGT1A9 played a primary role in glucuronidation in human liver microsomes (HLM; $K(m) = 81.3$ μM, $V(max) = 242.2$ pmol/min/mg protein). In addition, both mycophenolic acid (substrate of UGT1A9) and emodin (substrate of UGT1A8 and UGT1A10) could inhibit the glucuronidation of Picroside II with the half maximal inhibitory concentration (IC(50))

values of 173.6 and 76.2 μM, respectively. Enzyme kinetics was also performed in HIMs. The K(m) value of Picroside II glucuronidation was close to that in recombinant human UGT1A10 (K(m) = 58.6 μM, V(max) = 721.4 pmol/min/mg protein). The intrinsic clearance was 5.4-fold of HLMs. Intestinal UGT enzymes play an important role in Picroside II glucuronidation in human.

Title: Effect of picroside II on expressions of TLR4 and NFkappaB in rats with cerebral ischemia reperfusion injury

PMID: 21434346

Publication Date: 2011 Jan

Gist: Picroside II could down-regulate the expressions of TLR4 and NFkappaB, and inhibit the inflammatory response induced apoptosis in cerebral I/R injured rats.

Title: Neuroprotective properties of picroside II in a rat model of focal cerebral ischemia.

PMID: 21151457

Publication Date: 2010 Nov 16

Gist: The aim of this study was to explore the effect of picroside II on neuronal apoptosis and the expression of caspase-3 and poly ADP-ribose polymerase (PARP) following middle cerebral artery occlusion/reperfusion in male Wistar rats. Caspase-3 and PARP expressions were also profound in the cortex, the striatum and the hippocampus, along with increased apoptotic cells in this group. Bederson's score, infarction volume, and expressions of caspase-3 and PARP, as well as apoptosis in the treatment group were, however, significantly decreased compared to those in the control group indicating that intravenous treatment with picroside II might be beneficial to inhibit neuronal apoptosis and, thus, to improve the neurological function of rats upon cerebral ischemia reperfusion injury.

Title: Development of novel lipidated analogs of picroside as vaccine adjuvants: acylated analogs of picroside-II elicit strong Th1 and Th2 response to ovalbumin in mice.

PMID: 20688035

Publication Date: 2010 Dec 6

Gist: The acylated analogs of picroside-II were synthesized and tested for immune-adjuvant activity in the presence of weak antigen ovalbumin found to stimulate anti-OVA IgG titer, neutralizing antibody (IgG1 and IgG2a) titer as well as the production of soluble mediators of a Th1 response (IL-2 and IFN-γ) and Th2 response (IL-4) and proliferation of T lymphocytes sub-sets (CD4/CD8). Furthermore, these modified analogs of picroside-II were able to elicit a substantial increase in anti-OVA IgG when compared with OVA alone. These results support the use of acylated analogs particularly PK-II-3 and PK-II-4 as potent enhancer of antigen-specific Th1 and Th2 immune responses and thus are promising immune-adjuvant candidate for vaccines.

Title: Anti-inflammation effects of picroside 2 in cerebral ischemic injury rats.

PMID: 20618938

Publication Date: 2010 Jul 9

Gist: Picrodide 2 could inhibit neuronal apoptosis and play anti-oxidant and anti-inflammation role in cerebral ischemia/reperfusion injuries, but the exact mechanism is not very clear. This study aims to explore the anti-inflammation mechanism of picroside 2 in cerebral ischemic reperfusion injury in rats. Picroside 2 could down-regulate the expressions of TLR4, NFkappaB and TNFalpha to inhibit apoptosis and inflammation induced by cerebral ischemic reperfusion injury and improve the neurobehavioral function of rats.

Title: Antinociceptive and anti-inflammatory effects of saponin and iridoid glycosides from Verbascum pterocalycinum var. mutense Hub.-Mor.

PMID: 18274283

Publication Date: 2007 Nov

Gist: Although antinociceptive and anti-inflammatory activities of ajugol and picroside IV were found insignificant in the statistical analysis.

Title: Synergistic protective effect of picroside II and NGF on PC12 cells against oxidative stress induced by H2O2.

PMID: 18048958

Publication Date: 2007 Sep

Gist: Epidemiological studies suggest that nerve growth factor (NGF) is associated with a reduced risk of acute or chronic neuropathies. We studied the synergistic protective effect of picroside II and NGF against the oxidative stress in PC12 cells induced by hydrogen peroxide (H2O2). The fluorescent probe CDCFH was used to assess the intracellular reactive oxygen species (ROS) level, and MTT assay, morphological observation as well as LDH leakage test were conducted to measure cellular injury. The H2O2-induced cytotoxicity was significantly attenuated in the presence of picroside II (25 microg/ml) and NGF (2 ng/ml). Cultures with this combined treatment possessed decreased level of ROS while increased cell survival, as compared to that of picroside

II or NGF alone-treated cells. Accordingly, it was concluded that their synergistic protective activities against oxidative stress in vitro were demonstrated in various aspects, including reversing morphological changes, enhancing the ability of cell proliferation and ROS scavenging. Such action supports the therapeutic potential of picroside II and NGF in treating nervous disorders based on their synergistic effect.

Title: The neuroprotective effect of picroside II from hu-huang-lian against oxidative stress.

PMID: 17708634

Publication Date: 2007

Gist: To evaluate the neuroprotective effect of picroside II, PC12 cells were treated with glutamate in vitro and male ICR mice were treated with AlCl(3) in vivo. Pre-treatment of PC12 cells with

picroside II could enhance the cell viability and decrease the level of intracellular reactive oxygen species (ROS) induced by glutamate. By DNA fragmentation and flow cytometry assay, picroside II (1.2 mg/ml) significantly prevented glutamate-induced cell apoptosis. In the animal study, amnesia was induced in mice by AlCl(3) (100 mg/kg/d, i.v.). Pricroside II, at the dose of 20 and 40 mg/kg/d (i.g.), markedly ameliorated AlCl(3)-induced learning and memory dysfunctions and attenuated AlCl(3)-induced histological changes. This was associated with the significant increased superoxide dismutase (SOD) activity in the brain of experimental mice. All these results indicated that picroside II possessed the therapeutic potential in protecting against neurological injuries damaged by oxidative stress.

Title: Anti-lipid peroxidation and protection of liver mitochondria against injuries by picroside II.

PMID: 15968718

Publication Date: 2005 Jun 28

Gist: To investigate the anti-lipid peroxidation and protection of liver mitochondria against injuries in mice with liver damage by picroside II. Picroside II can evidently relieve hepatocyte injuries induced by CCl(4), D-GalN and AP, help scavenge free radicals, protect normal constructions of mitochondria membrane and enhance the activity of ATPase in mitochondria, thereby modulating the balance of liver energy metabolism, which might be part of the mechanisms of hepatoprotective effects of picroside II.

Title: Inhibitory effect of picroside II on hepatocyte apoptosis.

PMID: 15916740

Publication Date: 2005 Jun 26

Gist: To investigate the influence of picroside II on hepatocyte apoptosis and its mechanism. Picroside II can protect hepatocytes against injury and prevent hepatocytes from apoptosis. It might by upregulating the bcl-2 gene expression and antioxidation.

Title: Picrosides I and II, selective enhancers of the mitogen-activated protein kinase-dependent signaling pathway in the action of neuritogenic substances on PC12D cells.

PMID: 12151059

Publication Date: 2002 Aug 30

Gist: Picrosides I and II caused a concentration-dependent (> 0.1 microM) enhancement of basic fibroblast growth factor (bFGF, 2 ng/ml)-, staurosporine (10 nM)- and dibutyryl cyclic AMP (dbcAMP, 0.3 mM)-induced neurite outgrowth from PC12D cells. PD98059 (20 microM), a potent mitogen-activated protein (MAP) kinase kinase inhibitor, blocked the enhancement of bFGF (2 ng/ml)-, staurosporine (10 nM)- or dbcAMP (0.3 mM)-induced neurite outgrowth by picrosides, suggesting that picrosides activate MAP kinase-dependent signaling pathway. However, PD98059 did not affect the bFGF (2 ng/ml)-, staurosporine (10 nM)- and dbcAMP (0.3 mM)-induced neurite outgrowth in PC12D cells, indicating the existence of two components in neurite outgrowth induced by bFGF, staurosporine and dbcAMP, namely the MAP kinase-independent and the masked MAP kinase-dependent one. Furthermore, picrosides-induced enhancements of the bFGF-action were

markedly inhibited by GF109203X (0.1 microM), a protein kinase C inhibitor. The expression of phosphorylated MAP kinase was markedly increased by bFGF (2 ng/ml) and dbcAMP (0.3 mM), whereas that was not enhanced by staurosporine (10 nM). Picrosides had no effect on the phosphorylation of MAP kinase induced by bFGF or dbcAMP and also unaffected it in the presence of staurosporine. These results suggest that picrosides I and II enhance bFGF-, staurosporine- or dbcAMP-induced neurite outgrowth from PC12D cells, probably by amplifying a down-stream step of MAP kinase in the intracellular MAP kinase-dependent signaling pathway. Picrosides I and II may become selective pharmacological tools for studying the MAP kinase-dependent signaling pathway in outgrowth of neurites induced by many kinds of neuritogenic substances including bFGF.

Title: Nerve growth factor-potentiating compounds from Picrorhizae Rhizoma.

PMID: 10919373

Publication Date: 2000 Jul

Gist: The ethyl acetate solubles were fractionated by silica gel chromatography, monitoring the NGF-potentiating activity to give two iridoid glycosides, picrosides I and II. Picrosides did not exhibit neurotrophic activity but caused a marked enhancement of the NGF-mediated neurite outgrowth from PC12D cells. The pharmacological data suggest that picrosides I and II enhance neurite outgrowth from PC12D cells, probably by amplifying a step in the NGF-receptor-mediated intracellular signaling pathway.

Title: Picrorhiza kurroa (Kutaki) Royle ex Benth as a hepatoprotective agent--experimental & clinical studies.

PMID: 9715310

Publication Date: 1996 Oct

Gist: Picrorhiza kurroa (Pk), a known hepatoprotective plant, was studied in experimental and clinical situtations. The standardization of active principles--Picroside 1 and 2 was done with High Performance Liquid Chromatography. Picroside 1 ranged from 2.72 to 2.88 mg/capsule and picroside 2 from 5.50 to 6.00 mg/capsule. In the galactosamine-induced liver injury in rats, Pk at a dose of 200 mg/kg p.o. showed a significant reduction ($p < 0.05$) in liver lipid content, GOT and GPT. In a randomised, double-blind placebo controlled trial in patients diagnosed to have acute viral hepatitis (HBsAg negative), Pk root powder 375 mg three times a day was given for 2 weeks ($n = 15$) or a matching placebo ($n = 18$) was given. Difference in values of bilirubin, SGOT and SGPT was significant between placebo and Pk groups. The time in days required for total serum bilirubin to drop to average value of 2.5 mg% was 75.9 days in placebo as against 27.44 days in Pk group. The present study has shown a biological plausability of efficacy of Pk as supported by clinical trial in viral hepatitis, hepatoprotection in animal model and an approach for standardizing extracts based on picroside content.

Title: Picroliv and its components kutkoside and picroside I protect liver against galactosamine-induced damage in rats.

PMID: 1333078

Publication Date: 1992 Nov

Gist: Oral administration of Picroliv (12 mg/kg/day for 7 days), a standardised iridoid glycoside fraction of Picrorhiza kurroa, significantly prevented the biochemical changes in liver and serum of galactosamine-toxicated rats. Kutkoside (12 mg/kg/day for 7 days) also protected against changes in most of the hepatic and serum constituents studied. Another iridoid glycoside from Picroliv, Picroside I, at the same dose level could only prevent toxicant-induced changes in acid phosphatase, phospholipids and lipid peroxides in liver and alkaline phosphatase in serum. Mixture of Picroside I and Kutkoside in the ratio of 1:1.5 at 12 mg/kg dose elicited lesser response than Picroliv.

Title: Picroliv, picroside-I and kutkoside from Picrorhiza kurrooa are scavengers of superoxide anions.

PMID: 1321626

Publication Date: 1992 Jul 7

Gist: Picroliv, the active principle of Picrorhiza kurrooa, and its main components which are a mixture of the iridoid glycosides, picroside-I and kutkoside, were studied in vitro as potential scavengers of oxygen free radicals. The superoxide (O2-) anions generated in a xanthine-xanthine oxidase system, as measured in terms of uric acid formed and the reduction of nitroblue tetrazolium were shown to be suppressed by picroliv, picroside-I and kutkoside. Picroliv as well as both glycosides inhibited the non-enzymic generation of O2- anions in a phenazine methosulphate NADH system. Malonaldehyde (MDA) generation in rat liver microsomes as stimulated by both the ascorbate-Fe2+ and NADPH-ADP-Fe2+ systems was shown to be inhibited by the Picroliv glycosides. Known antioxidants tocopherol (vitamin E) and butylated hydroxyanisole (BHA) were also compared with regard to their antioxidant actions in the above system. It was found that BHA afforded protection against ascorbate-Fe(2+)-induced MDA formation in microsomes but did not interfere with enzymic or non-enzymic O2- anion generation; and tocopherol inhibited lipid peroxidation in microsomes by both prooxidant systems and the generation of O2- anions in the non-enzymic system but did not interfere with xanthine oxidase activity. The present study shows that picroliv, picroside-I and kutkoside possess the properties of antioxidants which appear to be mediated through activity like that of superoxide dismutase, metal ion chelators and xanthine oxidase inhibitors.

Title: In vitro studies on the effect of certain natural products against hepatitis B virus.
PMID: 2370093

Publication Date: 1990 Apr

Gist: Picroliv (active principle from Picrorrhiza kurroa), its major components picroside I, catalpol, kutkoside I, kutkoside, andrographolide (active constituent of Andrographis paniculata), silymarin and Phyllanthus niruri extract were tested for the presence of anti hepatitis B virus surface antigen (anti HBs) like activity. HBsAg positive serum samples obtained from hepatitis B virus (HBV) associated acute and chronic liver diseases and healthy HBsAg carriers were used

to evaluate the anti-HBs like activity of compounds/extract. The latter were mixed with serum samples and incubated at 37 degrees C overnight followed by HBsAg screening in the Elisa system. A promising anti-HBsAg like activity was noted in picroliv (and its major components) catalpol, P. niruri which differed from the classical viral neutralization. Picroliv also inhibited purified HBV antigens (HBsAg and HBsAg) prepared from healthy HBsAg carriers. The in vitro testing system appears to be a suitable model to identify an agent active against HBV, prior to undertaking detailed studies.

Title: Protection against Amanita phalloides by the iridoid glycoside mixture of Picrorhiza kurroa (kutkin).

PMID: 2339676

Publication Date: 1990 Mar

Gist: Survival of mice after lethal doses of lyophilizate from Amanita phalloides ('death cap') was markedly increased by pretreatment with single doses of kutkin, a mixture of iridoid glycosides picroside I and kutkoside isolated from the roots of Picrorhiza kurroa. The protective effect of kutkin was comparable to that of silibinin. The curative efficacy of kutkin appeared to be slightly superior.

Title: Iridoid glycosides-Kutkin, Picroside I, and Kutkoside from Picrorrhiza kurroa Benth inhibits the invasion and migration of MCF-7 breast cancer cells through the down regulation of matrix metalloproteinases : 1st Cancer Update

DOI: 10.1016/j.arabjc.2011.01.011

Publication Date: 2011 Jan

Gist: MCF-7 cell lines (Human breast cancer) were used to test whether P. kurroa extract (PE) and its isolated iridoid glycosides Picroside I (PS), Kutkoside (KS), and Kutkin (KT) exerts the anti-invasion activity via down-regulation of the expression of matrix metalloproteinases (MMPs). MMPs play an important role in solid tumor invasion and migration.

Title: Recent advances in herbal medicine for treatment of liver diseases. [Review Article]

PMID: 21595500

Publication Date: 2011 Sep

Gist: Picroliv has been found to possess potent hepatoprotective activity against different hepatotoxins (Dwivedi et al., 1993). Hepatoprotective activity of picroliv has been established against thioacetamide (TAA) (Dwivedi et al., 1991), CCl (Dwivedi et al., 1990) and alcohol (Rastogi et al., 1996). CCl4 treatment resulted in elevation of serum ALT and AST and reduction of liver GSH, G6PD, catalase and membrane-bound Na4+/K ATPase which was reversed by administration of Picroliv. Thus, Picroliv offers significant protection against liver damage by

CCl+ by acting as free radical scavenger and inhibitor of LPO of liver plasma membrane (Santra et al., 1998).
